# Pathway-specific TNF-mediated metaplasticity in hippocampal area CA1

**DOI:** 10.1038/s41598-022-05844-1

**Published:** 2022-02-02

**Authors:** Anurag Singh, Shruthi Sateesh, Owen D. Jones, Wickliffe C. Abraham

**Affiliations:** Department of Psychology, Brain Health Research Centre, Brain Research New Zealand, University of Otago, Box 56, Dunedin, 9054 New Zealand

**Keywords:** Hippocampus, Tumour-necrosis factors, Long-term potentiation

## Abstract

Long-term potentiation (LTP) is regulated in part by metaplasticity, the activity-dependent alterations in neural state that coordinate the direction, amplitude, and persistence of future synaptic plasticity. Previously, we documented a heterodendritic metaplasticity effect whereby high-frequency priming stimulation in stratum oriens (SO) of hippocampal CA1 suppressed subsequent LTP in the stratum radiatum (SR). The cytokine tumor necrosis factor (TNF) mediated this heterodendritic metaplasticity in wild-type rodents and in a mouse model of Alzheimer’s disease. Here, we investigated whether LTP at other afferent synapses to CA1 pyramidal cells were similarly affected by priming stimulation. We found that priming stimulation in SO inhibited LTP only in SR and not in a second independent pathway in SO, nor in stratum lacunosum moleculare (SLM). Synapses in SR were also more sensitive than SO or SLM to the LTP-inhibiting effects of pharmacological TNF priming. Neither form of priming was sex-specific, while the metaplasticity effects were absent in TNFR1 knock-out mice. Our findings demonstrate an unexpected pathway specificity for the heterodendritic metaplasticity in CA1. That Schaffer collateral/commissural synapses in SR are particularly susceptible to such metaplasticity may reflect an important control of information processing in this pathway in addition to its sensitivity to neuroinflammation under disease conditions.

## Introduction

Long-term potentiation (LTP) and long-term depression (LTD) are widely accepted candidates as neural mechanisms underlying memory formation. Moreover, past neuronal state can regulate LTP and LTD via metaplasticity, the activity-dependent changes in neuronal state that regulate future synaptic plasticity, and that may participate in the maintenance of synaptic thresholds within normal limits^1,2^. In a unique form of metaplasticity in area CA1 of the rodent hippocampus, prior electrical activity (priming activity) in either stratum oriens (SO) or stratum radiatum (SR) inhibits subsequent LTP and promotes LTD at a separate set of synapses in SR. This heterosynaptic effect is independent of postsynaptic neuronal firing and activation of *N*-methyl-D-aspartate receptors (NMDARs), metabotropic glutamate receptors (mGluRs) and gamma-aminobutyric acid (GABA) GABA-A and –B receptors. Instead, the priming effect appears to involve activation of non-neural cells, such as astrocytes, across the hippocampal network^3,4^ Accordingly, we have hypothesized that gliotransmitters are released onto nearby synapses to cause the metaplastic effect.

Several cytokines, notably tumour necrosis factor (TNF) and interleukin-1 beta (IL-1β), suppress LTP in the CA1 region of the hippocampus^5–7^. Recently, we reported that TNF but not IL-1β mediates the long-range metaplastic inhibition of LTP in CA1 of rat hippocampus, while pharmacological priming by TNF but not IL-1β protein causes a similar inhibition of LTP^8^. Further, we reported that both the electrical and pharmacological TNF-mediated metaplastic effects involve activation of p38 mitogen-activated protein kinase (p38 MAPK), extracellular signal-regulated kinases (ERK) and c-Jun N-terminal kinases (JNK)^8^. However, the receptor subtype that TNF binds to in order to activate these kinases is unknown. TNF is a pleiotropic pro-inflammatory cytokine that binds to two receptor subtypes, TNF receptor I (TNFR1, p55) and TNF receptor II (TNFR2, p75). TNFR1 activation is associated with an enhanced glutamate receptor responsiveness^9,10^, neuropathic pain^11^, and the progression of AD^12^. Moreover, TNFR1 is required for the amyloid-beta (Aβ) mediated inhibition of LTP in the dentate gyrus^13–15^ and CA1^16^. Therefore, we hypothesised that TNF binding to TNFR1 is essential for the metaplastic inhibition of LTP in CA1.

Interestingly, Aβ causes inhibition of LTP in SR but not in SO^17,18^. This raises the question whether SR synapses are also more sensitive to TNF-mediated metaplasticity effects than SO synapses. Equally, it is unknown whether synapses in the third main dendritic layer of CA1, the stratum-lacunosum moleculare (SLM) that contains the pyramidal cell apical tuft dendrites, is more like SO or SR in its sensitivity to TNF-mediated inhibition of LTP. To address these questions, we compared the relative sensitivity of SO, SR and SLM synapses to electrical and TNF priming. Further, since all our prior studies used male animals, we compared the priming effects between males and females.

We found that the metaplasticity effect occurred differentially across strata, as electrical priming in SO only inhibited LTP in SR but not in SO or SLM. Similarly, SR synapses were more sensitive to TNF priming than SO or SLM synapses. Electrical and TNF priming effects in SR occurred in both females and males and required TNFR1 activation.

## Results

### Pathway-specific metaplasticity effects of HFS priming in stratum oriens

**Figure 1:**
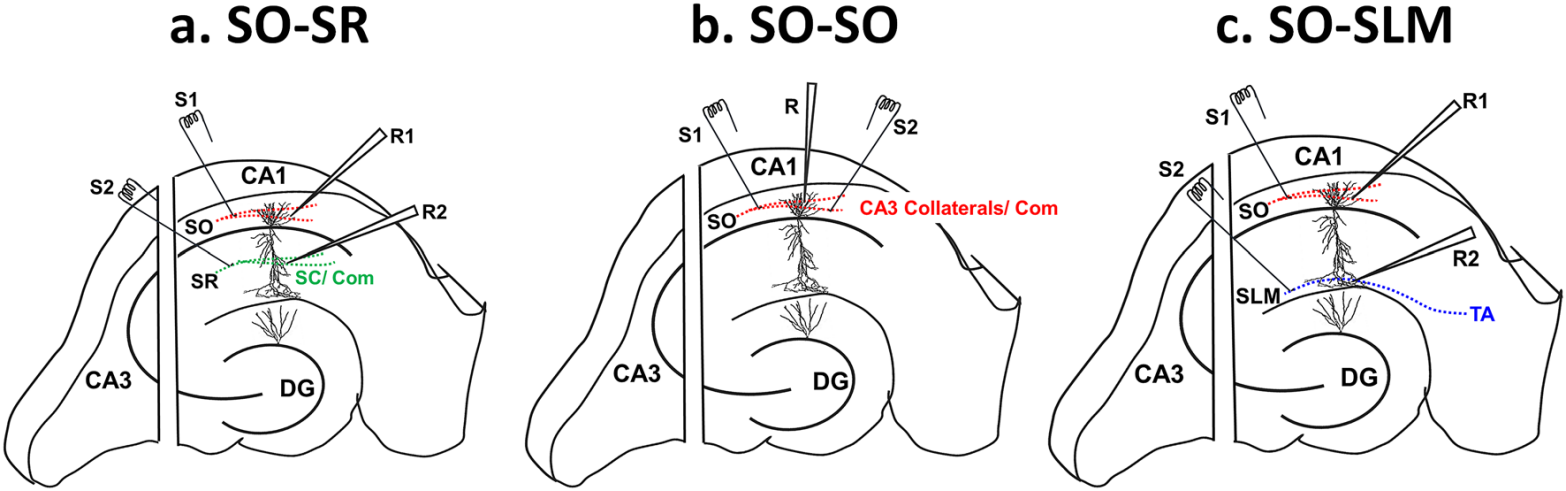
A Simplified schematic diagram of the hippocampus representing electrode placement in SR, SO and SLM. (**a**) SO-SR electrode placements. S1 and S2 represent stimulating electrodes, and R1 and R2 represent recording electrode placement in SO and SR, respectively. (**b**) SO-SO electrode placements. S1 and S2 represent the stimulation electrode placement on opposite ends in SO, while R represents the recording electrode placed at the centre of S1 and S2 (see Supplementary Fig. S1 for the test of pathway independence in SO). (**c**) SO-SLM electrode placements. S1 and S2 represent stimulating electrodes, and R1 and R2 represent recording electrode placements in SO and SLM, respectively. The pyramidal neuron image shows its processes extending from stratum pyramidale (SP) towards SR (apical dendrites) and SO (basal dendrites). (Com, commissural fibers; SC, Schaffer collaterals; TA, temporoammonic input; DG, dentate gyrus).

**Figure 2:**
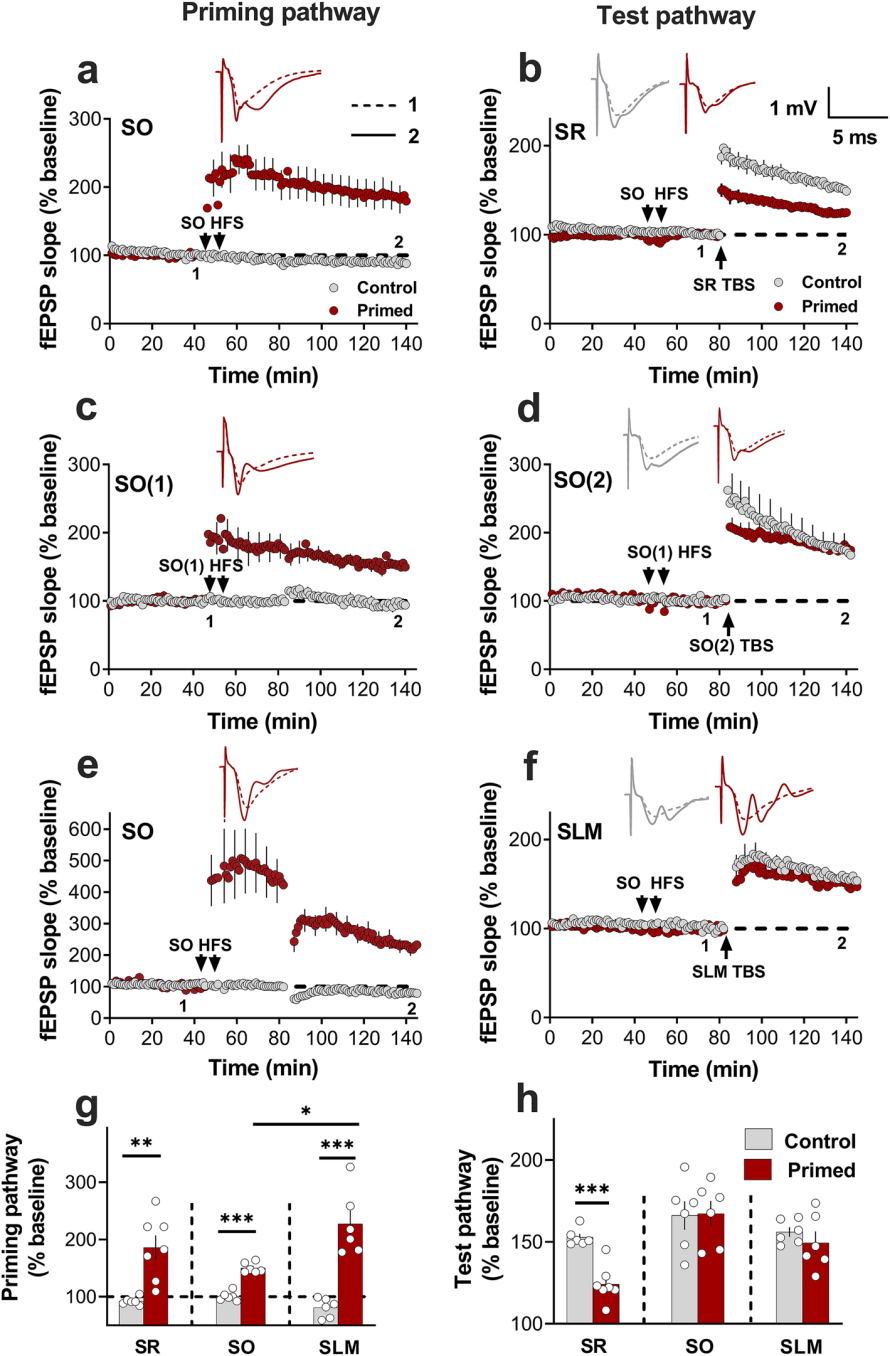
Pathway specificity of HFS priming in SO. (**a**) HFS priming produced significant LTP in the SO primed pathway compared to the control (non-priming) condition (Control = 89.8 ± 4%, n = 5; Primed = 185.9 ± 18%, n = 15; *t*_(10)_ = 3.7, *p* = 0.002), and (**b**) produced significant inhibition of LTP in the test SR pathway when compared to the control condition (Control = 152.5 ± 10%, n = 5; Primed = 124.3 ± 6%, n = 7; *t*_(10)_ = 5.12, *p* = 0.0004). (**c**) As in (**a**), HFS priming produced significant LTP in SO (Control = 100.6 ± 5%, n = 6; Primed = 150.4 ± 18%, n = 6; *t*_(10)_ = 10.7, *p* = 0.0001), but (**d**) had no effect on LTP at neighbouring synapses in the same stratum (Control = 170.5 + 12%, n = 6; Primed = 177.6 + 9%, n = 6; *t*_(10)_ = 0.2861, *p* = 0.78). (**e**) In the SLM experiment, HFS priming produced significant LTP in SO (Control = 81.07 ± 7%, n = 6; Primed = 227.3 ± 24%, n = 6; *t*_(10)_ = 13.03, *p* =  < 0.0001) but (**f**) had no effect on SLM LTP (Control = 156.6 ± 3%, n = 6; Primed = 149.4 ± 8%, n = 6; *t*_(10)_ = 0.88, *p* = 0.39). (**g**) Bar graph summarising the homosynaptic LTP in SO across all three experiments. The SO LTP in the SLM experiment was statistically different when compared to that in the SO experiment (*p* = 0.02) but not the SO LTP in the SR experiment (*p* = 0.243). (**h**) Bar graph summarising the electrical priming effects on LTP in SR, SO and SLM. For the inset waveforms in all figures, 1 represents the average of the final 10 sweeps taken before tetanization in the relevant pathway (pre) and 2 represents the average of 10 sweeps taken 50–60 min after TBS (post) of the test pathway. All data in this and other figures are presented as mean ± SEM; ^∗^, p < 0.05; ^∗∗^, p < 0.01; ^∗∗∗^, p < 0.001.

To assess the pathway specificity of the priming effect, we first sought to replicate the previously reported finding that priming inhibits subsequent LTP in SR, using male Sprague–Dawley rats^19^. In this paradigm, following a 45 baseline recording period, priming stimulation (6 trains of high-frequency stimulation (HFS) are given to the SO afferents and 30 min later, LTP is 30 induced in the Schaffer collateral afferents in SR through two trains of theta-burst stimulation (TBS, see Methods for specific details). In confirmation of our previous results, priming in SO (Figs. 1a, 2a,g) caused a significant inhibition of LTP in SR 30 min later (Student’s t-test; *p* = 0.0004; Fig. 2b,h). In contrast, the same priming stimulation in a separate group of animals (Figs. 1b, 2c,g) did not affect LTP in a second independent set of synapses in SO (Student’s t-test; *p* = 0.78; Fig. 2d,h; see Supplementary Fig. S1 for confirmation of pathway independence in SO). Interestingly, priming in SO (Figs. 1c, 2e,g) had no effect on LTP at synapses in SLM (Student’s t-test; *p* = 0.39; Fig. 2f,h). These pathway differences occurred in a different pattern to what was observed for LTP in the SO priming pathway across the three experiments (one-way ANOVA *F*_(2, 15)_ = 4.750, *p* = 0.025). Here, post-hoc tests revealed no differences between experiments except for a significantly greater SO LTP in the SLM experiment relative to that in the SO experiment (*p* = 0.02), likely because of the inclusion of GABA receptor antagonists throughout the SLM experiment (see Methods) to ensure adequate LTP induction in SLM under control conditions^20^.

### Pathway specificity of the metaplasticity effects of TNF priming

**Figure 3:**
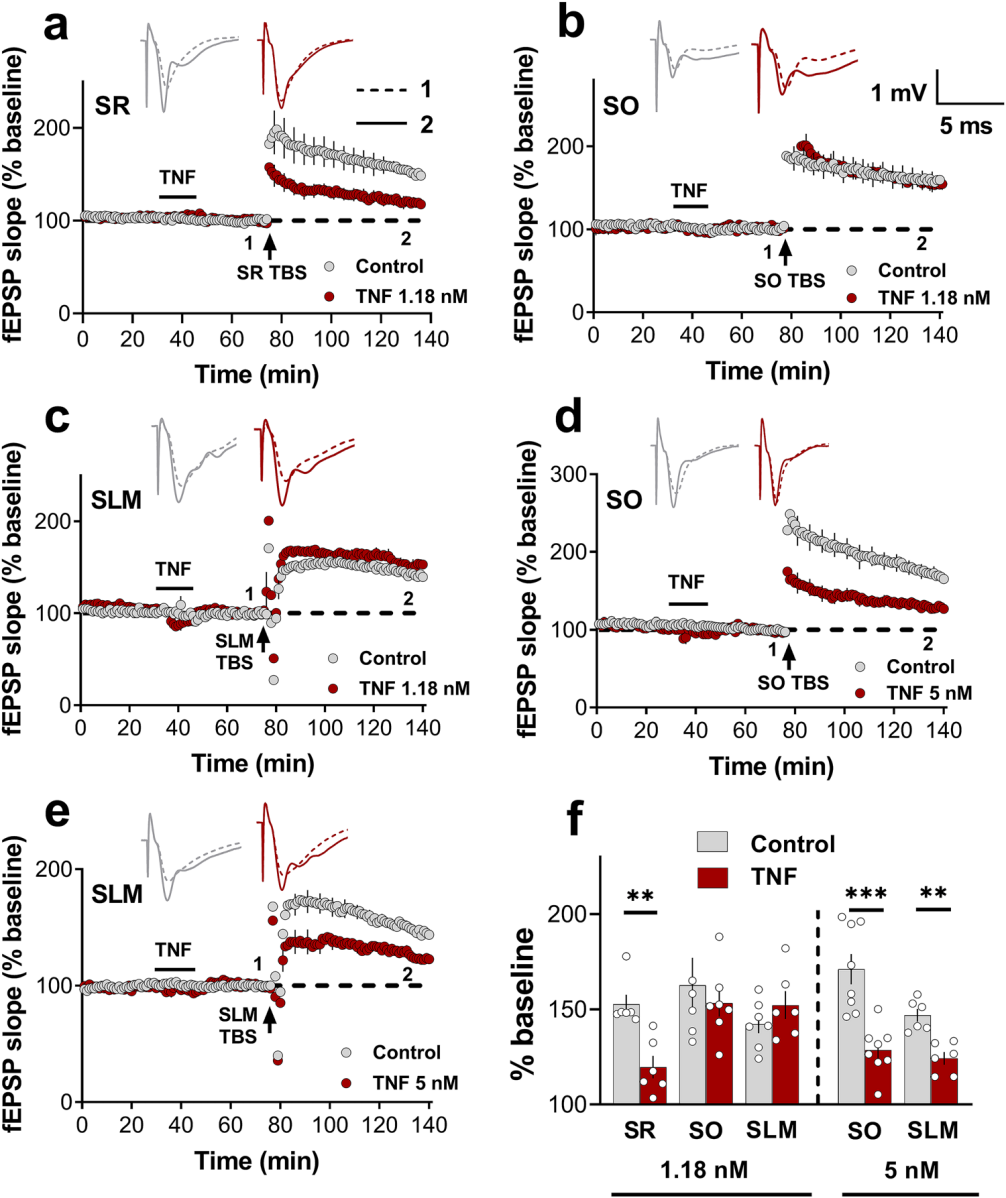
Pathway specificity of TNF priming. (**a**) Bath application of 1.18 TNF (bar) inhibited SR LTP (Control = 152.6 ± 7%, n = 6; TNF = 119.6 ± 5%, n = 6; *t*_(10)_ = 4.24, *p* = 0.001). (**b**) Bath application of TNF had no effect on SO LTP (Control = 165.1 ± 8%, n = 9; TNF = 153.3 ± 7%, n = 7; *t*_(14)_ = 0.90, *p* = 0.37), or (**c**) SLM LTP (Control = 154.7.1 ± 8%, n = 6; TNF = 152.2 ± 6%, n = 6; *t*_(10)_ = 0.26, *p* = 0.79). (**d**) At 5 nM, bath application of TNF inhibited SO LTP (Control = 171.1 ± 12%, n = 8; TNF = 128.6 ± 9%, n = 8; *t*_(14)_ = 4.61, *p* = 0.0004), and (**e**) SLM LTP (Control = 148 ± 7%, n = 8; TNF = 124.3 ± 4%, n = 5; *t*_(9)_ = 4.33, *p* = 0.001). (**f**) Bar graph summarising the differential effects of TNF on LTP in the different strata. ^∗∗^, p < 0.01; ^∗∗∗^, p < 0.001.

We next investigated whether the priming of LTP by bath application of TNF (1.18 nm) exhibited a similar pathway specificity. TNF at a low concentration (nM range) has been found to be effective in inhibiting LTP at SR synapses both when given concurrently^6^ and as a priming stimulus^8^. Here, a 10 min bath application of 1.18 nM TNF protein caused significant inhibition of LTP induced 30 min later in SR compared to ACSF controls (Student’s t-test; *p* = 0.001; Fig. 3a,f). Consistent with the electrical priming results above, the same TNF priming protocol in separate groups had no effect on SO LTP (Student’s t-test; *p* = 0.37; Fig. 3b,f) nor on SLM LTP (Student’s t-test; *p* = 0.28; Fig. 3c,f). The vehicle treatment (PBS + 0.1% BSA) by itself produced no effect on the LTP in SR, SO or SLM relative to ACSF controls (Supplementary Fig. S2). In SR and SO, the TNF treatment did not significantly affect the baseline field potentials up to the point of the LTP induction. However, in SLM, TNF treatment significantly decreased the baseline responses, but this was a transient effect that completely decayed after washing out the TNF for 20 min (Supplementary Fig. S3).

### Sufficient TNF does inhibit LTP at SO and SLM synapses

Although 1.18 nM TNF had no effect on LTP at the SO and SLM synapses, we asked whether this was a relative or absolute difference in TNF sensitivity. Accordingly, we tested the efficacy of a higher concentration of TNF in inhibiting subsequent LTP at these synapses. We found that 5 nM TNF was sufficient to inhibit LTP at SO synapses (Student’s t-test; *p* = 0.0004; Fig. 3d,f) to a similar extent as 1.18 nm TNF in SR. Likewise, LTP at SLM synapses was inhibited to a similar extent (Student’s t-test; *p* = 0.001; Fig. 3e,f). These findings indicate that the pathway specificity was a relative difference in TNF sensitivity, being greater at SR synapses than for SO and SLM synapses.

### TBS priming inhibits SR LTP

**Figure 4:**
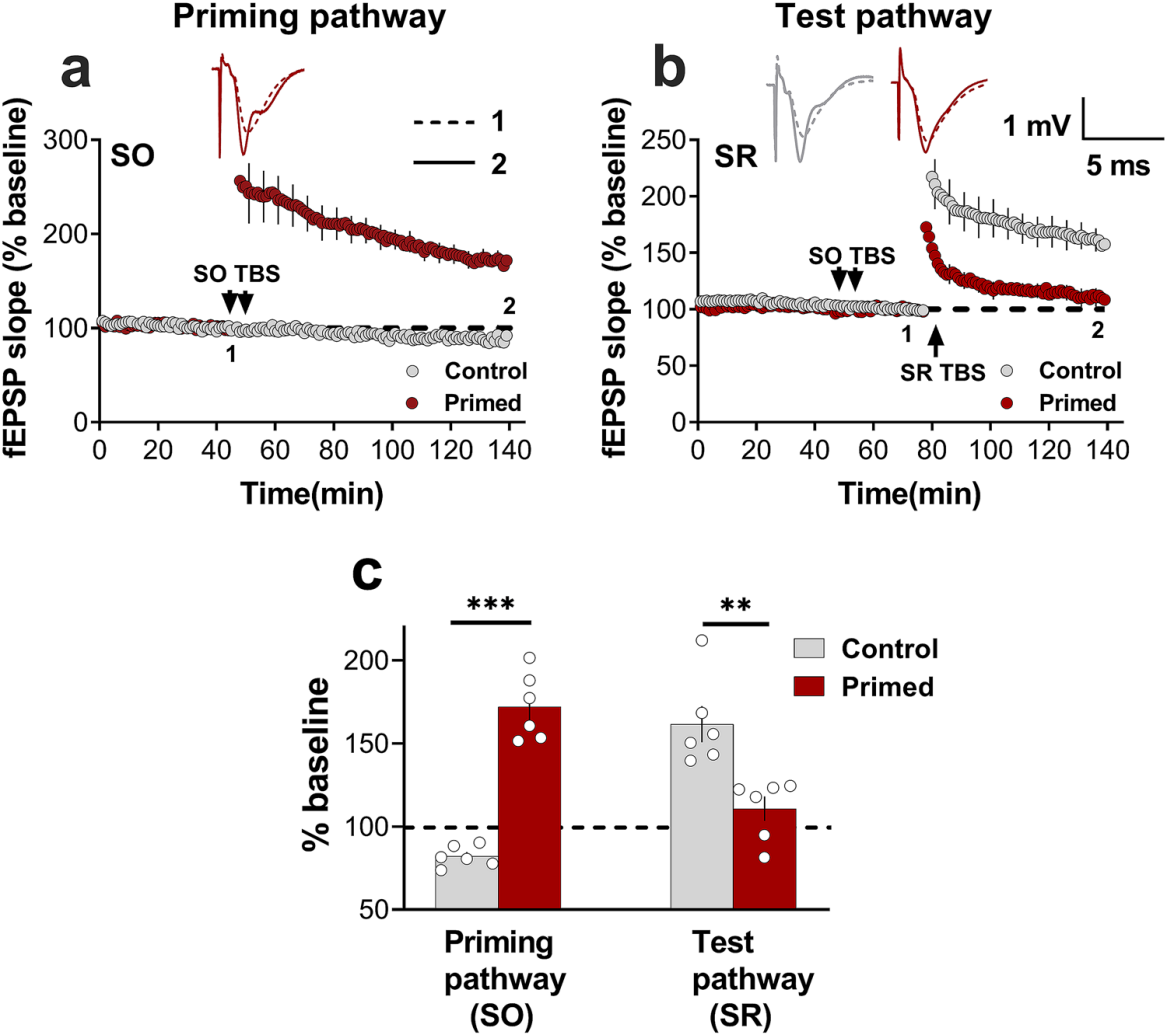
TBS priming inhibits SR LTP. (**a**) TBS priming produced substantial LTP in SO (Control = 88.8 ± 3%, n = 5; Primed = 166.4 ± 8%, n = 6; *t*_(10)_ = 8.1, *p* = 0.0001), and (**b**) significantly inhibited LTP in SR (Control = 161.5 ± 8%, n = 6; Primed = 110.7 ± 3%, n = 6; *t*_(10)_ = 3.86, *p* = 0.003). (**c**) Bar graph summarising the TBS-induced homosynaptic LTP in SO and inhibition of LTP in SR. ^∗∗^, p < 0.01; ^∗∗∗^, p < 0.001.

To date, our standard priming protocol has entailed 6 trains of HFS, as originally described^21^, although 4 trains have also been effective^3^. Since it is widely accepted that theta-burst stimulation is a more biological pattern of high-frequency neuronal activity, we investigated whether fewer stimuli at this pattern (100 pulses total compared to 400–600 pulses for HFS) could also generate the priming effect. Similar to HFS priming, two trains of TBS priming in SO not only produced homosynaptic LTP in SO (Fig. 4a,c) but also caused significant inhibition of LTP in SR (Student’s t-test; *p* = 0.003; Fig. 4b,c).

### Heterodendritic metaplasticity in SR requires TNFR1 in both male and female mice

TNF plays a critical role in mediating the heterodendritic metaplasticity effect in area CA1 of the rat hippocampus^8^. However, the receptor subtype involved in mediating the metaplasticity mechanisms remains unclear. As it has been reported that TNFR1 plays a role in other plasticity mechanisms^13,22^, we tested the hypothesis that TNFR1 is necessary for the heterodendritic metaplasticity effect using the TNFR1 deficient mouse model (TNFR1^−/−^).

**Figure 5:**
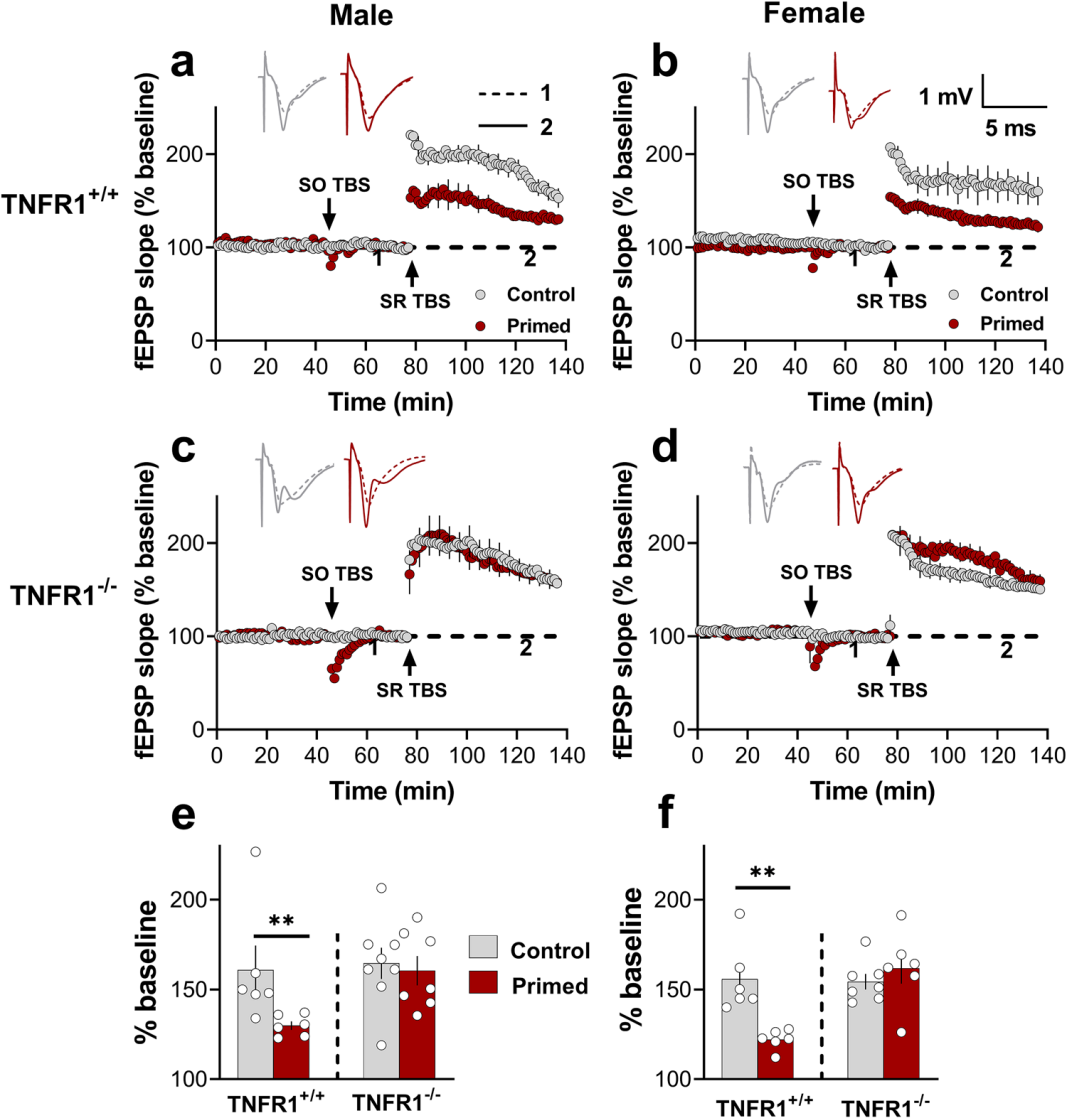
TBS priming-induced metaplasticity in SR requires TNFR1 in both male and female mice. (**a**) Tukey’s post-hoc analysis revealed that there was significant inhibition of LTP due to priming in the SR of TNFR1^+/+^ male mice (Control = 160.9 ± 15%, n = 6; Primed = 129.8 ± 8%, n = 6; *p* = 0.004), and (**b**) female mice (Control = 159.8 ± 13%, n = 6; Primed = 122.1 ± 9%, n = 6; *p* = 0.002, control vs. primed). (**c**) In contrast, the priming effect was lacking in the SR of male TNFR1^-/-^ mice (Control = 160.5 ± 13%, n = 7; Primed = 164.6 ± 12%, n = 8; *p* = 0.98) and (**d**) female TNFR1^-/-^ mice (Control = 154.4 ± 7%, n = 7; Primed = 161.9 ± 9%, n = 6; *p* = 0.91) mice. (**e**) Histogram summarizing the electrical priming effect in SR of male TNFR1^+/+^ but not TNFR1^-/-^ mice. (**f**) Histogram summarizing the electrical priming effect in SR of female TNFR1^+/+^ but not TNFR1^-/-^ mice. Two-way ANOVA on male and female TNFR1^+/+^ animals revealed a significant main effect for experimental group [*F*_(1,20)_ = 12.05, *p* =  < 0.0001] but no significant main effect of gender [*F*_(1,20)_ = 0.12, *p* = 0.72] or interaction [*F*_(1,20)_ = 0.007, *p* = 0.93], and two-way ANOVA on male and female TNFR1^-/-^ revealed no significant main effect for experimental group [*F*_(1,24)_ = 0.55, *p* = 0.82], gender [*F*_(1,24)_ = 0.32, *p* = 0.57], or interaction [*F*_(1,24)_ = 0.04, *p* = 0.82]. ^∗∗^, p < 0.01.

TBS priming in SO inhibited SR LTP in male TNFR1^+/+^ mice (two-way ANOVA; *p* = 0.004; Fig. 5a,e), similar to the effect reported above in male rats (Fig. 4b) and previously in male C57Bl/6 mice^3^. The TBS priming also readily inhibited LTP in female TNFR1^+/+^ mice (two-way ANOVA; *p* = 0.006; Fig. 5b,f). In contrast, the TBS-mediated priming effect was absent in both male (two-way ANOVA; *p* = 0.98; Fig. 5c,e) and female (two-way ANOVA; *p* = 0.91; Fig. 5d,f) TNFR1^−/−^ mice. Despite these differences, there was no difference in the amount of LTP generated in SO by the priming stimulation for male and female TNFR1^+/+^ compared to the TNFR1^-/-^ mice (Supplementary Fig. S4). We also performed electrical priming experiments in heterozygous TNFR1 (TNFR1^+/−^) mice. Similar to TNFR1^−/−^ mice, priming stimulation in TNFR1^+/−^ mice produced a significant homosynaptic LTP in SO, but in this case a partial priming-mediated inhibition of LTP in SR (Student’s t-test; *p* = 0.043; Supplementary Fig. S5).

### TNF-induced metaplasticity in SR requires TNFR1

**Figure 6:**
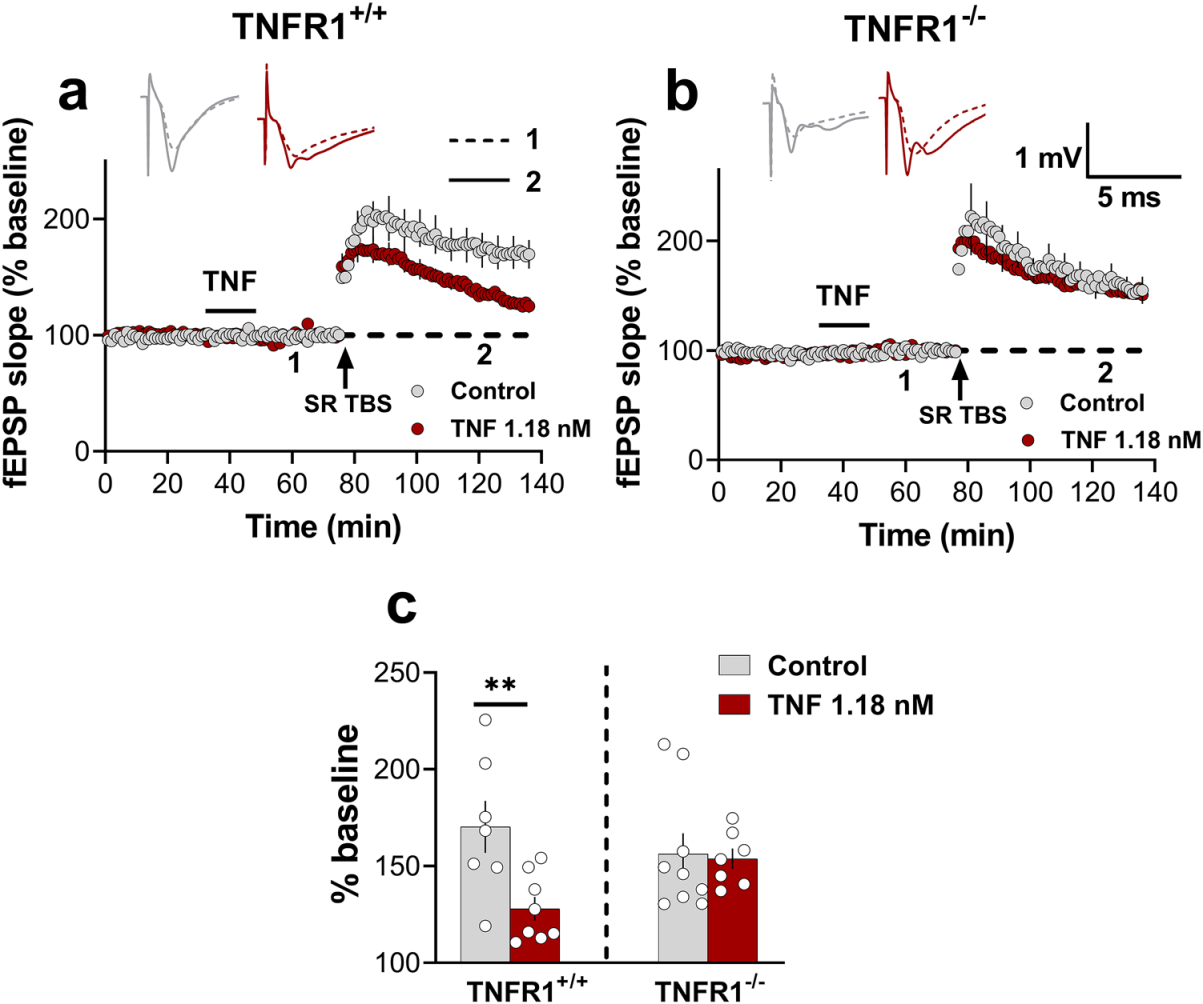
TNF-induced metaplasticity in SR requires TNFR1. (**a**) Bath application of 1.18 nM TNF significantly inhibited SR LTP in TNFR1^+/+^ male and female mice (Control = 170.3 ± 14%, n = 7; TNF = 128 ± 9%, n = 8; *t*_(13)_ = 2.99, *p* = 0.01). (**b**) TNF had no effect on the LTP in TNFR1^-/-^ male and female mice (Control = 156.3 ± 10%, n = 9; TNF = 153.8 ± 13%, n = 7; *t*_(14)_ = 0.19, *p* = 0.84). (**c**) Bar graph summarising the TNF-mediated priming effect in TNFR1^+/+^ mice but not in TNFR1^-/-^ mice. ^∗∗^, p < 0.01.

Since 1.18 nM TNF was sufficient to produce the metaplasticity effect in SR of the rat hippocampus^8^ (Fig. 3a), we next investigated whether this effect was also dependent on TNFR1. Given that the heterodendritic metaplasticity effect in SR was not sex-specific, we combined the sexes for these experiments, with roughly equal numbers of males and females in each group. We found that TNF at this concentration produced a substantial inhibition of later LTP in TNFR1^+/+^ mice (Student’s t-test; *p* = 0.01; Fig. 6a,c), but not in TNFR1^-/-^ mice (Student’s t-test; *p* = 0.84; Fig. 6b,c), confirming a critical role for TNFR1 in mediating the metaplastic inhibition of LTP in SR of CA1.

## Discussion

LTP is vital for learning and memory and is regulated in part by metaplasticity. In one particularly interesting form of metaplasticity, priming stimulation in SO, the basal dendritic zone of CA1 pyramidal cells, inhibits LTP induction 30 min later at Schaffer collateral/commissural synapses located a few hundred microns away in the apical dendritic zone in SR^3,19^. Here we asked whether LTP at other CA1 input pathways was equally affected. Interestingly, the same SO priming protocol that impaired SR LTP had no effect on LTP in either a second SO pathway or in SLM. Thus, there is differential regulation of synaptic plasticity in sub-regions of CA1. The lack of a priming effect in SO is intriguing because the excitatory afferents of both SR and SO are Schaffer collateral and commissural fibres from CA3. The inputs to SLM, in contrast, arise from layer 3 pyramidal cells in the entorhinal cortex (the medial entorhinal cortex, given that our SLM stimulation was delivered in proximal CA1^23^). However, the finding in SO is consistent with our previous report that priming stimulation in SR, which also inhibits SR LTP, failed to inhibit LTP in SO^19^.

Recently we reported that TNF release mediates the long-range metaplasticity effect between the SO and SR dendritic compartments^8^. Moreover, delivery of recombinant TNF to slices mimicked the priming effect elicited by SO stimulation^8^, supporting previous studies using similar paradigms^5,6,24,25^. Here, we confirmed the role of TNF in priming-mediated inhibition of LTP and showed that it acts via the TNFR1 receptor, as both the electrical and TNF priming effects were absent in TNFR1^−/−^ mice. Notably, this was true for both male and female mice, demonstrating a lack of sex differences in the priming effect generally and the TNF mechanism in particular. The present experiments also showed the TNF concentration that is sufficient to inhibit SR LTP failed to affect either SO or SLM LTP, corresponding well with the sensitivity of SR synapses to the electrical priming effect. On the other hand, TNF delivered at the higher concentration of 5 nM could inhibit SO and SLM LTP. Thus, these strata have a higher threshold for TNF-mediated LTP inhibition compared to SR. This is consistent with the inhibition by amyloid-β of SR but not SO LTP, through a TNFR1-dependent mechanism^13^.

What could explain the sensitivity of SR synapses to the metaplasticity effect, especially relative to SO synapses that nominally have the same glutamatergic afferent inputs? Some clues may come from the anatomical, physiological and molecular differences between these pathways. For example, although there are indeed Schaffer collateral/commissural inputs to both SR and SO, the cells of origin are not entirely overlapping as SR receives its projections preferentially from proximal CA3 ipsilaterally, while SO receives its projections more from distal CA3 and contralateral CA3^26,27^. Moreover, CA2 pyramidal cells also project mainly to SO^28^. Postsynaptically, the basal and apical dendrites of CA1 pyramidal cells differ in size, shape and geometry, electrical properties, and responsiveness to neurotrophic factors^29–31^. In addition, dendrites in SO exhibit more mushroom spines and a higher magnitude of LTP than SR dendrites^32^. It is also possible that the pyramidal cell dendrites in SO and SLM with their bushy dendritic arbours may more readily generate synaptically evoked NMDAR spikes and plateau potentials^33,34^, which may make LTP induction there particularly resistant to counteracting influences. Mechanistically, it is notable that participation of nitric oxide (NO) is necessary for SR LTP but not for SO LTP, indicating a differential molecular mechanism of LTP expression in apical versus basal dendrites in CA1^35^. This may be due to the absence of endothelial NO synthase in SO, which is the primary source of NO in SR^36,37^. On the other hand, the enhancement of LTP in SO relative to SR is dependent on the expression of Type II cadherins^32^.

SLM synapses also display synaptic properties different from other hippocampal synapses. For example, in the developing hippocampus, the electrical stimulation that produces substantial LTD at SR synapses is less effective at SLM synapses^38^. In addition, the temporoammonic pathway displays distinct plasticity properties in response to the same stimulation strength (paired-pulse ratio, LTD) compared to Schaffer collateral/commissural synapses *in vivo*^39^. Conceivably, there could also be a differential regulation of TNF activity in SLM and SO compared to SR due to variations in TNFR1 distribution or activation threshold. However, we have not been able to detect differences in TNFR1 distribution across the CA1 strata, nor in the expression of downstream enzymes such as p38 MAP kinase or JUN kinase, using immunofluorescence imaging techniques (Sateesh et al., unpublished observations).

The mechanisms by which electrical priming and TNF act to inhibit SR LTP have yet to be fully characterised. Our previous experiments showed that this is not due to altered GABAergic inhibition, nor to altered NMDAR currents in SR^19^, which might otherwise explain why the suppression effect becomes evident so early post-tetanisation. Moreover, the priming stimulation in SO does not affect baseline transmission in SR except for a transient depression on occasion, indicating that a presynaptic change in transmitter release is unlikely to explain the effect. The inhibitory mechanism does rely on activation of p38 MAP kinase, JUN kinase and extracellular signal-related kinase^8^. However, the subcellular localisation of these kinases and their actions remains to be determined, especially given that astrocytes appear to also play a role in mediating this metaplasticity effect^3,4^.

TNF is produced in the CNS by neurons, astrocytes, and microglia^40^, and plays a prominent but paradoxical role in the brain because of its differential regulatory actions in healthy conditions versus during neurological disease or injury. TNF treatment on primary hippocampal neurons prevents hypoxia and NO-induced neuronal death^41^, ameliorates hippocampal damage in acute brain insults^42^ and stimulates synthesis and secretion of nerve growth factors^43^. In addition to a role in neuroprotection, as mentioned above, TNF has emerged as a regulator of synaptic function in the hippocampus, such as the trafficking of GABA-A^44^ and AMPA receptors^45^. Additionally, TNF acting on TNFR1 is critical for low-frequency stimulation-induced LTD in CA1 SR^46^ and metabotropic glutamate receptor-mediated LTD in the dentate gyrus^13^. Here we have confirmed an additional role for TNF, namely the pathway-specific metaplasticity evoked by both HFS and the more physiologically relevant TBS protocol.

What could be the behavioural relevance of the sensitivity of SR synapses to prior activity in either SR or SO afferents? SR inputs arise mainly although not exclusively from proximal CA3, where auto-associative connections mediate pattern completion and memory storage that is passed to area CA1 to consolidate activity there, such as place cell activity^47^. The output connections of CA1 with subicular and cortical regions are important for the replay of this information through the hippocampal and cortical circuitry and retrieval during memory retention trials as triggered by output from CA3^48^. Since the metaplasticity effect decays over a 1.5–2.5 h period, it is conceivable that the initial period of memory storage and consolidation is accompanied by a subsequent transient period of reduced memory storage functionality, i.e. LTP induction, to prevent interference from later information coming via CA3 while still allowing potential updating based on current information arising via the direct entorhinal inputs to SLM. The affected information could be particularly relevant to non-spatial recognition memory, mediated preferentially by the proximal CA3 input that projects to distal CA1 SR^49^. Note that the duration of the metaplasticity effect at physiological temperatures in vivo could be rather shorter than that noted by Holland and Wagner *in vitro*^50^.

TNF may deviate from its functions in healthy conditions at constitutive levels versus its level in a diseased condition. At physiologically relevant concentrations, TNF plays a role in controlling the strength of glutamatergic synapses in the hippocampal neurons^51^, mediates synaptic scaling in cortical synapses^52^, and participates in experience-dependent synaptic plasticity in the developing mouse visual cortex^53^. On the other hand, at higher concentrations, TNF inhibits LTP in both CA1 and the dentate gyrus of the hippocampus^5,6,24,54^. High levels of TNF have been detected in multiple sclerosis^55^, Alzheimer's disease (AD)^56^, and Parkinson's disease^57^. Moreover, animal and human studies indicate a correlation between increased TNF levels with cognitive and memory impairments^58,59^. Consistent with this, we have reported that the elevated TNF level in an APP/PS1 mouse model of AD generates an aberrant engagement of the heterodendritic metaplasticity mechanism responsible for the LTP impairment in these mice^8^, consistent with the efficacy of a scavenger biologic (XPro1585) in rescuing LTP in a 5xFAD mouse model^60^. Moreover, genetic and pharmacological block of TNFR1 prevents inflammation and cognitive impairment in APP/PS1 mice^61^ and less susceptibility to hippocampal epileptic seizure^9^. Together these data indicate a major role for TNF acting on TNFR1 in mediating cognitive impairment in neurodegenerative disease and suggest TNF as a therapeutic target.

In summary, CA1 pyramidal cells have a finely tuned sensitivity of LTP to prior activity in the network that differs between afferent pathways and dendritic compartments. This differential sensitivity may play an important role in the efficiency and consolidation of memory storage. Endogenous TNF acting on TNFR1 plays a fundamental role in mediating these pathway-specific heterodendritic metaplasticity effects. Maintaining the TNF concentration at an optimum level may be essential for synaptic homeostasis in the brain, while activity-dependent and transient elevations may play a critical role in regulating CA1’s learning, memory and retrieval functions.

## Material and methods

### Animals

Adult Sprague–Dawley (SD) male rats (6–8 weeks), and male and female mice (12–35 weeks) with a functional knock-out of the tumor necrosis factor 1 receptor^62^ (The Jackson Laboratory, Bar Harbor, USA, Tnfrsf1atm1Mak/J, Stock No: 002818) and their wild-type (WT) C57BL/6 littermates were used in this study. All of the animals were bred in specific-pathogen-free colonies at the University of Otago Breeding Station. Once delivered to the laboratory, the rats were kept in standard open-top cages (3–5 animals per cage), while in a different room, the mice were group-housed (2–3 animals per cage) in individually ventilated cages after weaning. All animals were given PuraWool (Purabed, Switzerland) as nesting material and acclimatized to the vivarium for at least one week prior to use. They were monitored daily with health status recorded on approved monitoring sheets. The light/dark cycle and ambient temperature were kept at 23 ± 2 °C, and 12:12 h, respectively, with the lights turned on at 6 a.m. The University of Otago Animal Ethics Committee approved the study objectives and design, as well as all animal handling and manipulation techniques, which were carried out in accordance with New Zealand animal welfare legislation. The study was designed in accordance with ARRIVE guidelines.

To produce the TNFR1^+/−^ mice (heterozygous), male TNFR1-KO mice obtained from The Jackson Laboratory were bred with female C57BL/6 mice present in our facility. TNFR1^+/−^ mice were crossed with TNFR1^+/−^ mice to produce TNFR1^+/+^ (wild type), TNFR1^+/−^, and TNFR1^−/−^ mice. To check the genotype of the animals, the DNA was isolated from mouse tail samples using the lysis buffer (25 mM NaOH and 40 mM Tris–Cl, pH 8.8). The DNA isolated was subjected to polymerase chain reaction (PCR) (conditions, denaturation 94 °C, annealing 65 °C and extension 72 °C), using the primers for TNFR1^+/+^ (WT, 5′–3′: TGT GAA AAG GGC ACC TTT ACG GC), TNFR1^+/−^ (Heterozygote, 5′–3′ GGC TGC AGT CCA CGC ACT GG) and TNFR1^−/−^ (Mutant, 5′–3′ATT CGC CAA TGA CAA GAC GCT GG) purchased from Invitrogen. To analyse and visualise the amplicons resulting from PCR, 1% agarose gel was prepared along with the addition of ethidium bromide (EtBr) at a final concentration of 0.5 μg/ml, the samples were loaded on to the precast wells followed by separation at 100 V for 1 h. The gel was exposed to UV light and a picture was taken using the gel documentation system (Gel Doc XR + Gel Documentation System, BioRad). The band appearing at 470 bp was identified as TNFR1^+/+^, 300 bp as TNFR1^−/−^, while bands appearing at both 470 bp and 300 bp as TNFR1^+/−^. The genotyping was done in-house for the first three generations and for subsequent generations the mouse tail tips were outsourced to Transnetyx (TN, USA) (see Supplementary Fig. S6 for molecular confirmation of genotypes).

### Slice preparation

Male Sprague–Dawley rats (n = 79) were deeply anaesthetized with ketamine (100 mg/kg, i.p.). Rats were decapitated, and brains were quickly removed and immersed in ice-cold sucrose cutting solution (mM: 210 sucrose, 26 NaHCO_3_, 2.5 KCl, 1.25 NaH_2_PO_4_, 0.5 CaCl_2_, 3 MgCl_2_, 20 d-glucose) continuously gassed with carbogen (95% O_2_–5% CO_2_). Hippocampi were dissected free, and the ventral portions removed. CA3 was removed manually with a knife cut to separate CA1 connectivity from the rest of the hippocampus. Using a Leica VT1000 S vibroslicer, 400 μm thick slices (3–6 slices from each hemisphere) were prepared. Slices were placed in a humidified incubation chamber with artificial cerebrospinal fluid (ACSF) and incubated at 32 °C (ACSF mM: 124 NaCl, 3.2 KCl, 1.25 NaH_2_PO_4_, 26 NaHCO_3_, 2.5 CaCl_2_, 1.3 MgCl_2_, 10 d-glucose) and gassed with carbogen. Before beginning the recordings, the slices were left at the interface between ACSF and humidified air for 30 min at 32 °C and then at room temperature for 90 min to equilibrate. Following incubation, up to two slices at a time were immersed in a humidified recording chamber at a temperature of 32.5 °C ± 0.5 °C and perfused with ACSF at 2–2.5 ml/min.

Male and female TNFR1^+/+^ (n = 47), TNFR1^−/−^ (n = 52), and TNFR1^+/−^ (n = 23) mice were given open-drop isoflurane anaesthesia (1 ml) in a closed desiccation jar before decapitation via guillotine. Horizontal brain sections of 400 μm thickness containing the hippocampus were prepared from each hemisphere. Mouse slices were then treated as per the rat slices, except that area CA3 was left intact. The experimenters were blind to genotype during data collection and analysis using a coding sheet held by a third party, with sex and genotype being quasi-randomly interspersed.

### Slice electrophysiology

To activate synaptic inputs and generate field excitatory postsynaptic potentials (fEPSPs), 50 μm Teflon-insulated tungsten monopolar stimulating electrodes were used, connected to custom-built programmable constant current stimulators. Stimulating electrodes were placed centrally in CA1 stratum radiatum (SR), stratum oriens (SO), and/or stratum lacunosum moleculare (SLM) to elicit fEPSPs (Fig. 1). Evoked fEPSPs were recorded using glass capillary micropipettes (1.0 mm × 0.58 mm, A-M Systems, WA, USA) pulled using P-97 Flaming/Brown micropipette puller (Sutter Instrument Company, CA, USA), coupled to Grass® P511 A.C. amplifiers with half-amplitude filter cut-offs of 0.3 kHz, 1–3 kHz. Recording electrodes were filled with ACSF and placed 400 µm apart in the same stratum as the stimulating electrode.

Slices were accepted for use if separate stimulation of afferents in SO or SR elicited synaptic fEPSPs with amplitudes greater than 1 mV in SO and 1.5 mV in SR, respectively, at a stimulation strength of 0.1 mA (0.1 ms half-wave duration). Similarly, the temporoammonic (TA) pathway was activated to elicit at least 2 mV field responses at a stimulation strength of 0.1 mA in SLM. Baseline stimulation was then set at 40% of the maximum fEPSP slope elicited at a stimulation strength of 0.2 mA. Baseline stimulation was delivered every 30 s, alternating between the priming stimulating electrode placed in SO and the test stimulating electrode in either SR, SO or SLM. For the TNF priming experiments, baseline stimulation was delivered every 30 s to the pathway of interest.

Control experiments included 75–80 min of baseline stimulation followed by LTP induction and 60 min post-tetanization recording. As an a priori criterion, slices which exhibited changes in baseline responses > 10% were excluded from the study. SO LTP was induced by a single train of theta-burst stimulation (TBS), each train consisting of 10 bursts of 5 pulses at 100 Hz, 200 ms between bursts, at baseline stimulation amplitude; in SR, the same stimulation was repeated twice with 30 s between the trains. The LTP stimulation protocol for SLM required four trains of TBS (with 10 pulses in each burst and pulse duration of 200 µs) with 30 s between trains. Because of the strength of synaptic inhibition in SLM, LTP induction in SLM also necessitated the bath application of a mixture of a GABA-A receptor antagonist (0.2 µM gabazine) and a GABA-B receptor antagonist (2 µM CGP 55845 hydrochloride). Thus, the LTP induction protocols were adjusted to deliver approximately the same degree of control LTP in each pathway.

We used two different electrical priming protocols in SO: either 2 sets of 3 trains of high-frequency stimulation (HFS, 100 Hz, 1 s, delivered 20 s apart, each set separated by 5 min)^8^ or 2 trains of theta-burst stimulation (TBS, as described above for SR LTP induction), 30 min before LTP induction in the test pathway. TNF protein priming entailed bath application of exogenous TNF for 10 min, with a washout period of 30 min before LTP induction^8^. Recordings from control and primed slices generated from the same animal were quasi-randomly interspersed throughout each experimental day. For the priming experiments, TNF protein (recombinant rat TNF-alpha protein; 510-RT R&D Systems) was reconstituted at 10 μg/mL in filter-sterilized PBS containing 0.1% bovine serum albumin as a vehicle. In this study, we used rats for the pathway specificity and the TBS priming experiments whereas mice were used for the investigation of TNF receptor subtype involved.

### Data analysis

FEPSPs were recorded in real-time and analyzed offline using custom-built software based on the LabVIEW library. The initial slopes of fEPSPs were measured, and the baseline value was calculated as the average fEPSP slope for the 10 min preceding LTP induction. All synaptic responses throughout the experiment were then expressed as a percentage of baseline. LTP was calculated by averaging the responses generated in the final 10 min of the experiment. The data for each group were pooled across animals with individual slices being the experimental unit of analysis (n’s). Group sizes were n = 5–9, as based on our previous studies of this metaplasticity effect^8^. A Grubb’s test was used to discard slices with outlier LTP values. The graphs were plotted, and statistical analyses (Student's t-tests and analyses of variance) were performed using GraphPad Prism software (version 9.2, San Diego, CA, USA). Tukey’s post-hoc analyses were utilized to find the difference between groups (significance set to p ≤ 0.05). All data are expressed as mean ± SEM unless otherwise stated.

### Ethics approval and consent to participate

All methods and procedures were approved by the University of Otago Animal Ethics Committee under animal ethics number AUP-19-42 and AUP-18-51. All the experiments were performed by adhering to the ethically approved protocols and in accordance with the New Zealand Animal Welfare and Biosecurity Legislation.

## Supplementary Information

Supplementary Information.

## Data Availability

Individual data points are shown in the bar graphs, and data will be made available upon reasonable request to the corresponding author.

## References

[1] Abraham WC, Bear MF (1996). Metaplasticity: The plasticity of synaptic plasticity. Trends Neurosci..

[2] Abraham WC (2008). Metaplasticity: Tuning synapses and networks for plasticity. Nat. Rev. Neurosci..

[3] Jones OD, Hulme SR, Abraham WC (2013). Purinergic receptor- and gap junction-mediated intercellular signalling as a mechanism of heterosynaptic metaplasticity. Neurobiol. Learn. Mem..

[4] Hulme SR, Jones OD, Raymond CR, Sah P, Abraham WC (2014). Mechanisms of heterosynaptic metaplasticity. Philos. Trans. R. Soc. Lond. B Biol. Sci..

[5] Butler MP, O'Connor JJ, Moynagh PN (2004). Dissection of tumor-necrosis factor-alpha inhibition of long-term potentiation (LTP) reveals a p38 mitogen-activated protein kinase-dependent mechanism which maps to early-but not late-phase LTP. Neuroscience.

[6] Tancredi V (1992). Tumor necrosis factor alters synaptic transmission in rat hippocampal slices. Neurosci. Lett..

[7] Bellinger FP, Madamba S, Siggins GR (1993). Interleukin 1 beta inhibits synaptic strength and long-term potentiation in the rat CA1 hippocampus. Brain Res..

[8] Singh A, Jones OD, Mockett BG, Ohline SM, Abraham WC (2019). Tumor necrosis factor-α-mediated metaplastic inhibition of LTP is constitutively engaged in an Alzheimer's disease model. J. Neurosci..

[9] Balosso S (2005). Tumor necrosis factor-α inhibits seizures in mice via p75 receptors. Ann. Neurol..

[10] Balosso S (2009). Molecular and functional interactions between tumor necrosis factor-alpha receptors and the glutamatergic system in the mouse hippocampus: Implications for seizure susceptibility. Neuroscience.

[11] Nadeau S (2011). Functional recovery after peripheral nerve injury is dependent on the pro-inflammatory cytokines IL-1β and TNF: Implications for neuropathic pain. J. Neurosci..

[12] Li R (2004). Tumor necrosis factor death receptor signaling cascade is required for amyloid-beta protein-induced neuron death. J. Neurosci..

[13] Wang Q, Wu J, Rowan MJ, Anwyl R (2005). Beta-amyloid inhibition of long-term potentiation is mediated via tumor necrosis factor. Eur. J. Neurosci..

[14] He P (2007). Deletion of tumor necrosis factor death receptor inhibits amyloid beta generation and prevents learning and memory deficits in Alzheimer's mice. J. Cell Biol..

[15] McAlpine FE (2009). Inhibition of soluble TNF signaling in a mouse model of Alzheimer's disease prevents pre-plaque amyloid-associated neuropathology. Neurobiol. Dis..

[16] Walsh DM (2002). Naturally secreted oligomers of amyloid beta protein potently inhibit hippocampal long-term potentiation in vivo. Nature.

[17] Hu NW, Klyubin I, Anwyl R, Rowan MJ (2009). GluN2B subunit-containing NMDA receptor antagonists prevent Abeta-mediated synaptic plasticity disruption in vivo. Proc. Natl. Acad. Sci. USA.

[18] Zhao J (2018). Soluble abeta oligomers impair dipolar heterodendritic plasticity by activation of mGluR in the hippocampal CA1 region. iScience.

[19] Hulme SR, Jones OD, Ireland DR, Abraham WC (2012). Calcium-dependent but action potential-independent BCM-like metaplasticity in the hippocampus. J. Neurosci..

[20] Remondes M, Schuman EM (2003). Molecular mechanisms contributing to long-lasting synaptic plasticity at the temporoammonic-CA1 synapse. Learn. Mem..

[21] Wang H, Wagner JJ (1999). Priming-induced shift in synaptic plasticity in the rat hippocampus. J. Neurophysiol..

[22] Dellarole A (2014). Neuropathic pain-induced depressive-like behavior and hippocampal neurogenesis and plasticity are dependent on TNFR1 signaling. Brain Behav. Immunol..

[23] Ito HT, Schuman EM (2012). Functional division of hippocampal area CA1 via modulatory gating of entorhinal cortical inputs. Hippocampus.

[24] Cunningham AJ, Murray CA, O'Neill LA, Lynch MA, O'Connor JJ (1996). Interleukin-1 beta (IL-1 beta) and tumour necrosis factor (TNF) inhibit long-term potentiation in the rat dentate gyrus in vitro. Neurosci. Lett..

[25] Cumiskey D, Butler MP, Moynagh PN, O'Connor JJ (2007). Evidence for a role for the group I metabotropic glutamate receptor in the inhibitory effect of tumor necrosis factor-alpha on long-term potentiation. Brain Res..

[26] Ishizuka N, Weber J, Amaral DG (1990). Organization of intrahippocampal projections originating from CA3 pyramidal cells in the rat. J. Comp. Neurol..

[27] Witter, M. P. & Amaral, D. G. In *The Rat Nervous System (Third Edition)* (ed George Paxinos) 635–704 (Academic Press, 2004).

[28] Shinohara Y (2012). Right-hemispheric dominance of spatial memory in split-brain mice. Hippocampus.

[29] McAllister AK, Lo DC, Katz LC (1995). Neurotrophins regulate dendritic growth in developing visual cortex. Neuron.

[30] Hausser M, Spruston N, Stuart GJ (2000). Diversity and dynamics of dendritic signaling. Science.

[31] Arikkath J (2012). Molecular mechanisms of dendrite morphogenesis. Front. Cell Neurosci..

[32] Basu R (2017). Heterophilic type II cadherins are required for high-magnitude synaptic potentiation in the hippocampus. Neuron.

[33] Takahashi H, Magee JC (2009). Pathway interactions and synaptic plasticity in the dendritic tuft regions of CA1 pyramidal neurons. Neuron.

[34] Gordon U, Polsky A, Schiller J (2006). Plasticity compartments in basal dendrites of neocortical pyramidal neurons. J. Neurosci..

[35] Ivanova VO, Balaban PM, Bal NV (2021). Nitric oxide regulates GluA2-lacking AMPAR contribution to synaptic transmission of CA1 apical but not basal dendrites. Front. Synaptic Neurosci..

[36] Son H (1996). Long-term potentiation is reduced in mice that are doubly mutant in endothelial and neuronal nitric oxide synthase. Cell.

[37] O'Dell TJ (1994). Endothelial NOS and the blockade of LTP by NOS inhibitors in mice lacking neuronal NOS. Science.

[38] Ma R, Xiao M, Gustafsson B (2016). Labile glutamate signaling onto CA1 pyramidal cells in the developing hippocampus depends mechanistically on input pathway. Neuroscience.

[39] Aksoy-Aksel A, Manahan-Vaughan D (2013). The temporoammonic input to the hippocampal CA1 region displays distinctly different synaptic plasticity compared to the Schaffer collateral input in vivo: significance for synaptic information processing. Front. Synaptic Neurosci..

[40] Olmos G, Lladó J (2014). Tumor necrosis factor alpha: A link between neuroinflammation and excitotoxicity. Mediat. Inflamm..

[41] Tamatani M (1999). Tumor necrosis factor induces Bcl-2 and Bcl-x expression through NFkappaB activation in primary hippocampal neurons. J. Biol. Chem..

[42] Gary DS, Bruce-Keller AJ, Kindy MS, Mattson MP (1998). Ischemic and excitotoxic brain injury is enhanced in mice lacking the p55 tumor necrosis factor receptor. J. Cereb. Blood Flow Metab..

[43] Hattori A (1993). Tumor necrosis factor stimulates the synthesis and secretion of biologically active nerve growth factor in non-neuronal cells. J. Biol. Chem..

[44] Pribiag H, Stellwagen D (2013). TNF-α downregulates inhibitory neurotransmission through protein phosphatase 1-dependent trafficking of GABA(A) receptors. J. Neurosci..

[45] Stellwagen D, Beattie EC, Seo JY, Malenka RC (2005). Differential regulation of AMPA receptor and GABA receptor trafficking by tumor necrosis factor-alpha. J. Neurosci..

[46] Albensi BC, Mattson MP (2000). Evidence for the involvement of TNF and NF-kappaB in hippocampal synaptic plasticity. Synapse.

[47] Dong C, Madar AD, Sheffield MEJ (2021). Distinct place cell dynamics in CA1 and CA3 encode experience in new environments. Nat. Commun..

[48] Rajasethupathy P (2015). Projections from neocortex mediate top-down control of memory retrieval. Nature.

[49] Nakamura NH, Flasbeck V, Maingret N, Kitsukawa T, Sauvage MM (2013). Proximodistal segregation of nonspatial information in CA3: preferential recruitment of a proximal CA3-distal CA1 network in nonspatial recognition memory. J. Neurosci..

[50] Holland LL, Wagner JJ (1998). Primed facilitation of homosynaptic long-term depression and depotentiation in rat hippocampus. J. Neurosci..

[51] Beattie EC (2002). Control of synaptic strength by glial TNFalpha. Science.

[52] Steinmetz CC, Turrigiano GG (2010). Tumor necrosis factor-alpha signaling maintains the ability of cortical synapses to express synaptic scaling. J. Neurosci..

[53] Kaneko M, Stellwagen D, Malenka RC, Stryker MP (2008). Tumor necrosis factor-alpha mediates one component of competitive, experience-dependent plasticity in developing visual cortex. Neuron.

[54] Maggio N, Vlachos A (2018). Tumor necrosis factor (TNF) modulates synaptic plasticity in a concentration-dependent manner through intracellular calcium stores. J. Mol. Med. (Berl).

[55] Hofman FM, Hinton DR, Johnson K, Merrill JE (1989). Tumor necrosis factor identified in multiple sclerosis brain. J. Exp. Med..

[56] Sly LM (2001). Endogenous brain cytokine mRNA and inflammatory responses to lipopolysaccharide are elevated in the Tg2576 transgenic mouse model of Alzheimer's disease. Brain Res. Bull..

[57] Mogi M (2000). Caspase activities and tumor necrosis factor receptor R1 (p55) level are elevated in the substantia nigra from parkinsonian brain. J. Neural Transm. (Vienna).

[58] Belarbi K (2012). TNF-α protein synthesis inhibitor restores neuronal function and reverses cognitive deficits induced by chronic neuroinflammation. J. Neuroinflamm..

[59] Holmes C (2009). Systemic inflammation and disease progression in Alzheimer disease. Neurology.

[60] MacPherson KP (2017). Peripheral administration of the soluble TNF inhibitor XPro1595 modifies brain immune cell profiles, decreases beta-amyloid plaque load, and rescues impaired long-term potentiation in 5xFAD mice. Neurobiol. Dis..

[61] Steeland S (2018). Counteracting the effects of TNF receptor-1 has therapeutic potential in Alzheimer's disease. EMBO Mol. Med..

[62] Pfeffer K (1993). Mice deficient for the 55 kd tumor necrosis factor receptor are resistant to endotoxic shock, yet succumb to L. monocytogenes infection. Cell.

