# Supplementary material for: Pathway-specific TNF-mediated metaplasticity in hippocampal area CA1
Source: Sci Rep. 2022 Feb 2;12:1746. doi: 10.1038/s41598-022-05844-1 (PMC8810872; doi:10.1038/s41598-022-05844-1)
Supplement: Supplementary file 1 — Supplementary Information. [file 41598_2022_5844_MOESM1_ESM.pdf]

Supplementary information

PATHWAY-SPECIFIC TNF-MEDIATED METAPLASTICITY IN HIPPOCAMPAL AREA CA1

Anurag Singh*, Shruthi Sateesh*, Owen D. Jones, and Wickliffe C. Abraham

Department of Psychology, Brain Health Research Centre, Brain Research New Zealand,

University of Otago, Dunedin 9054, New Zealand

* Joint first authors

Correspondence:

Professor Wickliffe C. Abraham
Department of Psychology
University of Otago
Box 56, Dunedin, 9054
New Zealand
cliff.abraham@otago.ac.nz

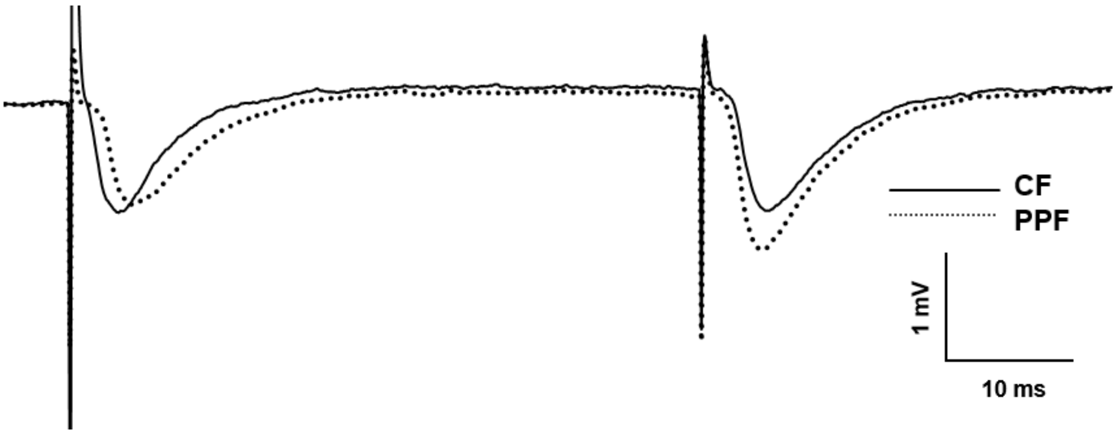

Supplementary Fig. 1. Representative paired-pulse facilitation (PPF) and cross-facilitation (CF) facilitation (PPF) waveforms indicating the stimulation of independent pathways in SO. To confirm pathway independence in SO, the paired-pulse ratio (50 ms inter-pulse interval) was determined separately for each stimulated pathway (dotted line), and then adjacent pathways were paired at the same interval (solid line). Only those experiments that exhibited paired-pulse facilitation in all stimulated pathways and no significant cross-facilitation after 5 repetitions were included.

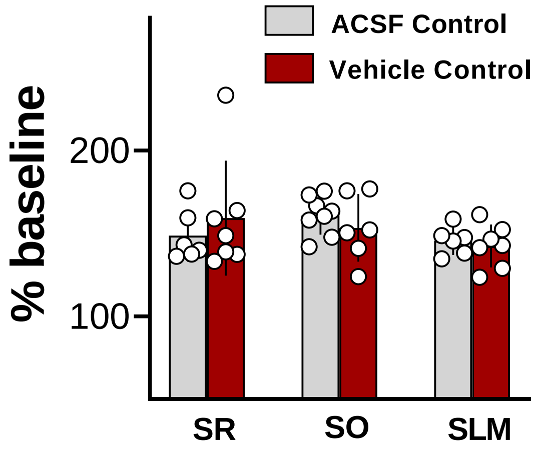

Supplementary Fig. 2. Comparison of LTP between ACSF and Vehicle controls in SR, SO and SLM experiments. Bath application of Vehicle (PBS+0.1% BSA) had no significant effect compared to ACSF controls on the TBS LTP in SR (ACSF = 148.6 $\pm$ 12%, n = 6; Vehicle = 159.1 $\pm$ 10%, n = 7; $t_{(11)}$ = 0.68, p = 0.50), SO (ACSF = 160.8 $\pm$ 8%, n = 8; Vehicle = 153.3 $\pm$ 9%, n = 6; $t_{(12)}$ = 0.88, p = 0.39), or SLM (ACSF = 145.5 $\pm$ 6%, n = 6; Vehicle = 142.4 $\pm$ 5%, n = 7; $t_{(11)}$ = 0.49, p = 0.62).

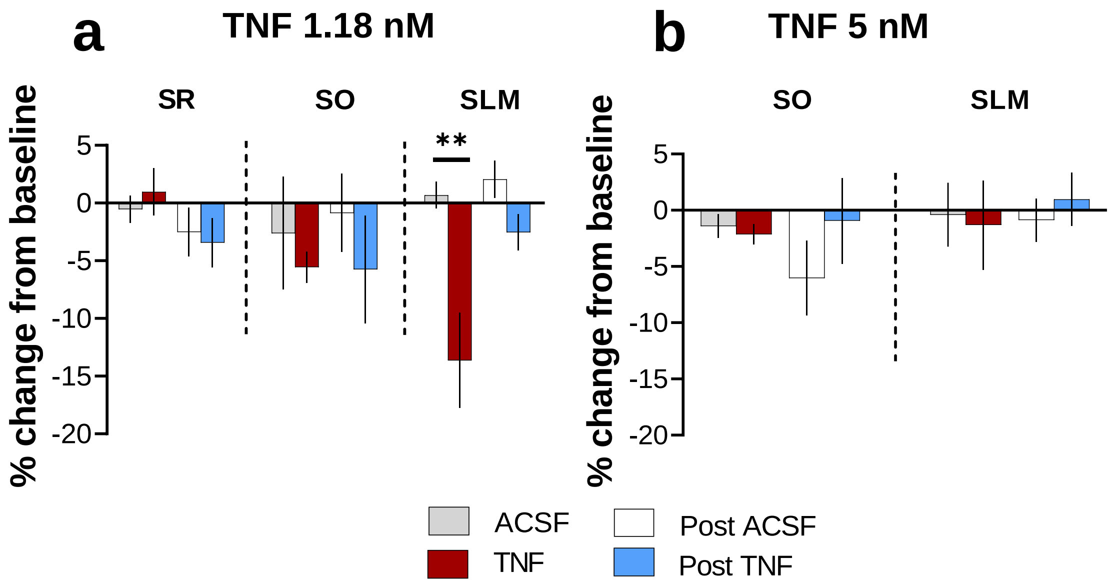

Supplementary Fig. 3. Effect of TNF treatment on baseline recordings in rats. (a) In SR, 1.18 nM TNF bath application had no effect on the baseline (% change from baseline: ACSF = -0.52%, n = 6; TNF = 0.97%, n = 6; $t_{(10)}$ = 0.63, p = 0.53). The % change in baseline after washing out the TNF or ACSF for 20 minute or more (post ACSF/TNF) was not statistically different (% change from baseline: Post ACSF = -2.5%, n = 6; Post TNF = -3.43%, n = 6; $t_{(10)}$ = 0.30, p = 0.76). Similarly in SO, TNF application had no effect on the baseline (% change from baseline: ACSF = -2.61%, n = 9; TNF = -5.56%, n = 7; $t_{(14)}$ = 0.51, p = 0.61). The % change after washing out TNF or ACSF was not statistically significant (% change from baseline: Post ACSF = -0.85%, n = 9; Post TNF = -5.75%, n = 7; $t_{(14)}$ = 0.87, p = 0.39). However, TNF at this concentration in SLM produced significant inhibitory effect (% change from baseline: ACSF = 0.67%, n = 6; TNF = -13.63%, n = 6; $t_{(10)}$ = 3.32, p = 0.007), The % change after washing out TNF or ACSF was not statistically significant (% change from baseline: Post ACSF = 2.04%, n = 6; Post TNF = -2.52%, n = 6; $t_{(10)}$ = 2.02, p = 0.07).. **(b)** At a higher concentration of 5nM, TNF had no effect on the baseline fEPSP values during the treatment in SO (% change from baseline- ACSF = -1.40%, n = 8; TNF = -2.14%, n = 8; $t_{(14)}$ = 0.53, p = 0.60). The % change after washing out TNF or ACSF was not statistically significant (% change from baseline: Post ACSF = -6.03%, n = 8; Post TNF = -0.93%, n = 8; $t_{(14)}$ = 1.01, p = 0.33). Similarly in SLM, 5nM TNF had no effect on the baseline fEPSP (% change from baseline- ACSF = -0.39%, n = 6; TNF = -1.31%, n = 5; $t_{(9)}$ = 0.19, p = 0.85). The % change after washing out TNF or ACSF was not statistically significant (% change from baseline: Post ACSF = -0.87%, n = 6; Post TNF = 0.96%, n = 5; $t_{(9)}$ = 0.6, p = 0.55). For the calculation, an average of 10 min before bath application of TNF/ACSF was used to calculate the baseline. This baseline was used to calculate the % change during ACSF or TNF treatment and after washout. **, p < 0.01.

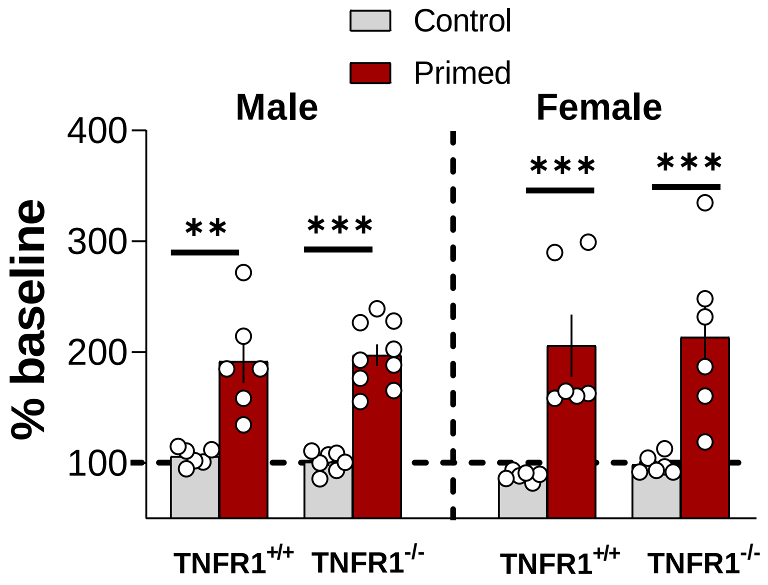

Supplementary Fig. 4. SO LTP due to electrical priming in male and female TNFR1$^{+/+}$ and TNFR1$^{-/-}$ mice. Two-way ANOVA on male and female TNFR1$^{+/+}$ data revealed a significant main effect for experimental group [$F_{(1,20)}$ = 34.87, p = <0.0001] but no main effect of gender [$F_{(1,20)}$ = 0.007, p = 0.93] or an interaction [$F_{(1,20)}$ = 0.86, p = 0.36]. Similarly, two-way ANOVA on male and female TNFR1$^{-/-}$ data revealed a significant main effect for experimental group [$F_{(1,24)}$ = 49.26, p = <0.0001] but no significant main effect of gender [$F_{(1,24)}$ = 0.12, p = 0.73] or an interaction [$F_{(1,24)}$ = 0.25, p = 0.61]. All data in this figure is presented as mean ± SEM; **, p < 0.01; ***, p < 0.001

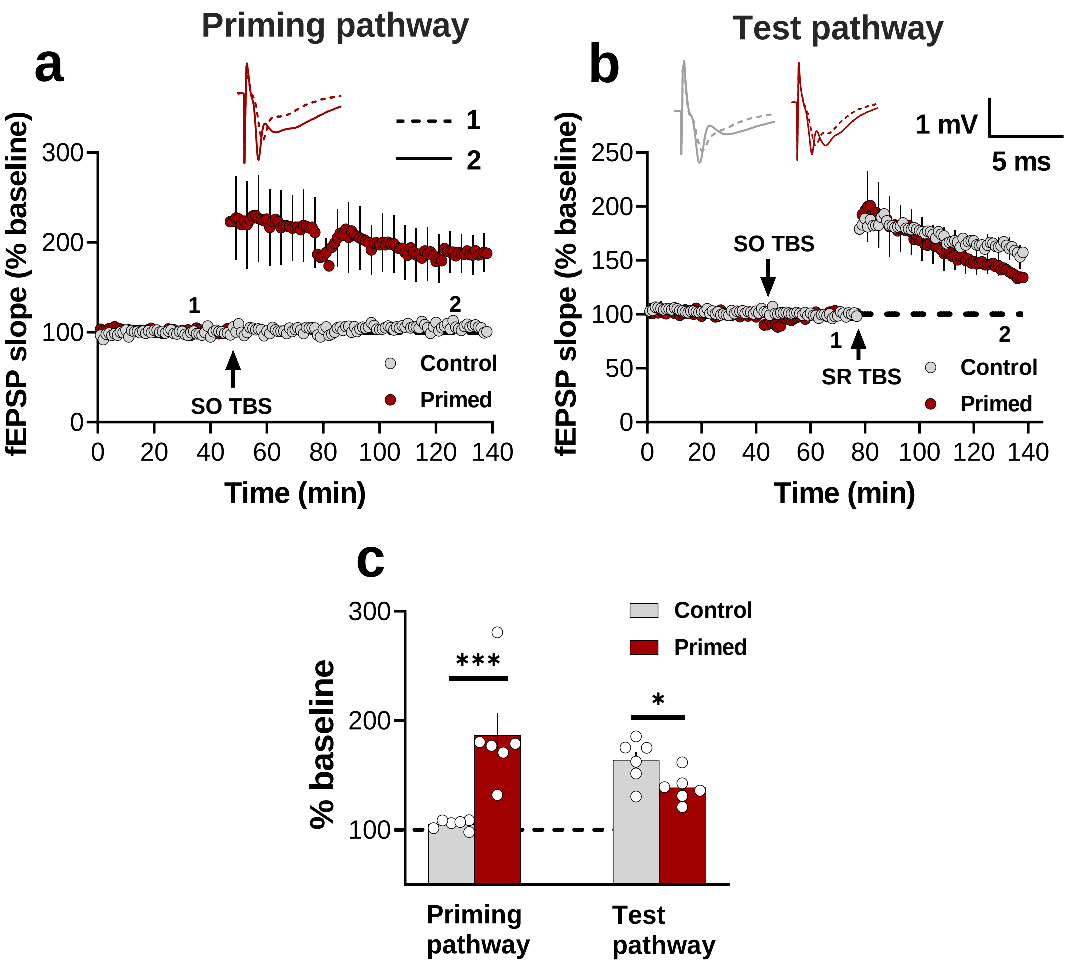

Supplementary Fig. 5. Heterozygous (TNFR1$^{+/-}$) animals show a partial priming effect in response to TBS priming. (a) In the priming pathway, priming in SO elicited homosynaptic LTP (Control = 105 $\pm$ 6%, n = 6; Primed = 186.5 $\pm$ 16%, n = 6; $t_{(10)}$ = 4.01, p = 0.002). **(b)** In the test pathway in SR, SO TBS priming partially inhibited LTP in SR when compared to control condition (Control = 163.4 $\pm$ 9%, n= 6; Primed = 138.6 $\pm$ 8%, n = 6; $t_{(10)}$ = 2.52, p = 0.043). **(c)** Bar graph summarising the induction of homosynaptic LTP in SO due to TBS priming and partial inhibition of SR LTP. *, p < 0.05; ***, p < 0.001.

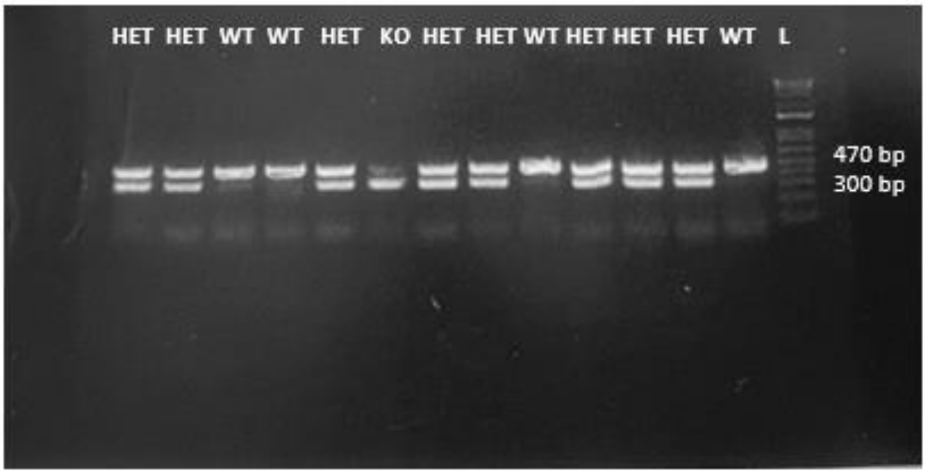

Supplementary Fig. 6. Molecular confirmation of the TNFR1$^{+/+}$, TNFR1$^{+/-}$ and TNFR1$^{-/-}$ genotypes. An auto-exposed gel showing extracted genomic DNA samples from mouse tail tips. The PCR products were directly loaded onto 1% agarose gel. Mutant or KO (TNFR1$^{-/-}$) band = 300 bp; WT (TNFR1$^{+/+}$) band = 470 bp; HET or Heterozygote (TNFR1$^{+/-}$) band = 300 bp and 470 bp (L = Loading Control).
